# Recent Development of Flexible and Stretchable Antennas for Bio-Integrated Electronics

**DOI:** 10.3390/s18124364

**Published:** 2018-12-10

**Authors:** Jia Zhu, Huanyu Cheng

**Affiliations:** 1 Department of Engineering Science and Mechanics, The Pennsylvania State University, University Park, PA 16802, USA; jmz5364@psu.edu; 2 Materials Research Institute, The Pennsylvania State University, University Park, PA 16802, USA

**Keywords:** flexible and stretchable antennas, textile, liquid metal, conductive composites, structural engineering

## Abstract

Wireless technology plays an important role in data communication and power transmission, which has greatly boosted the development of flexible and stretchable electronics for biomedical applications and beyond. As a key component in wireless technology, flexible and stretchable antennas need to be flexible and stretchable, enabled by the efforts with new materials or novel integration approaches with structural designs. Besides replacing the conventional rigid substrates with textile or elastomeric ones, flexible and stretchable conductive materials also need to be used for the radiation parts, including conductive textiles, liquid metals, elastomeric composites embedding conductive fillers, and stretchable structures from conventional metals. As the microwave performance of the antenna (e.g., resonance frequency, radiation pattern, and radiation efficiency) strongly depend on the mechanical deformations, the new materials and novel structures need to be carefully designed. Despite the rapid progress in the burgeoning field of flexible and stretchable antennas, plenty of challenges, as well as opportunities, still exist to achieve miniaturized antennas with a stable or tunable performance at a low cost for bio-integrated electronics.

## 1. Introduction

According to Maxwell’s equations, radiated fields are produced when a charge accelerates or decelerates [1]. Serving as an interface between radio waves propagating through space and electric currents moving in metal conductors, the antenna is the essential element of all radio equipment to convert between electric and electromagnetic energies. Chief performance measures of antennas are the directional characteristics as depicted in the radiation pattern and the resulting gain that accounts the efficiency. The other important parameters also include the resonance frequency and bandwidth. These properties are affected by the types of antennas, as well as the geometric and material parameters in each type. Though there are various antennas, the most widely studied ones are the monopole, dipole, and patch antennas, among others, due to their simple structure and ease of fabrication. 

As wireless technology plays a critical role in remote communication, non-contact charging (or powering), and identification, it is highly desirable to develop wearable antennas for the fast-developing flexible and stretchable electronic devices that have broad applications in health monitoring and clinic therapeutics [2,3,4]. As a representative example, radio-frequency identification (RFID) could wirelessly detect a fracture in a structure or the health condition of individuals from a relatively long distance without the need for an external power source [5,6]. In order to integrate electronics onto the human body, that is typically associated with curvilinear surfaces and dynamically changing motions, the bio-integrated devices must be conformal and physically flexible or even stretchable. As the key component in wireless technology, the representative patch antenna that consists of a rigid dielectric substrate and metal radiation parts in the traditional design is neither flexible nor stretchable. Because the bending stiffness of a thin film structure that characterizes its resistance against bending deformation roughly scales with the cubic of its thickness [7,8], thinning down the thickness of the structure represents an effective means to enable flexible/bendable antennas [9,10,11]. Due to the dictated thickness in the dielectric substrate, the rigid dielectric substrate is typically replaced by a flexible textile or elastomeric substrate in the design of the flexible antenna [12,13]. When the stretchable property is of concern for the antenna, approaches based on stretchable materials and/or stretchable structures have been explored. After replacing the rigid dielectric substrate with a flexible or stretchable substrate, the radiation parts of antennas can then be replaced by stretchable materials or be engineered into a stretchable layout. In either approach, a tradeoff between the stretchable mechanical property and the microwave performance of the system is observed [14,15]. In order to address this challenge, different strategies have been proposed and extensively studied. The radiation property of the flexible and stretchable antennas under mechanical deformation will be specifically discussed in this mini-review.

As the conductive component is the key for the radiation parts, the widely used methods to construct a conductive component over stretching have been studied for the stretchable antenna. In this mini-review, we will first discuss the considerations and implementations of flexible and stretchable antennas that are based on textiles. Next, we will introduce the composite elastomer with an interpenetrating network of liquid metal for the stretchable antenna. Composite elastomer embedding conductive fillers for the stretchable antenna will then be reviewed. Finally, we will briefly discuss the effort to explore stretchable structures from conventional metals for the stretchable antenna. Bearing the similar design principles as the patch antenna, the transmission lines that are essential to the input and output of electromagnetic signals will also be briefly discussed. Moreover, we will highlight the challenges and opportunities in the burgeoning field of flexible and stretchable antennas for future development.

## 2. Insulating and Conducting Fabrics for Textile Antennas

Due to the ease of integration on the clothes, flexible antennas that are based on textiles have attracted significant attention. In the textile antenna, the conventional dielectric substrate such as Rogers (dielectric constant of 3–10 and dielectric loss tangent of 0.001–0.005) is replaced by fabrics to enable flexibility. Depending on the properties of the fiber components and the structure of the yarns in the textile substrate, their dielectric constants and loss tangents are measured to be in the ranges of [1.5, 2] and [0.005, 0.05], respectively [16,17]. In comparison to the conventional substrate such as Rogers, the relatively low dielectric constant of textiles could provide a slightly large impedance bandwidth and a high radiation efficiency in the resulting antenna, but the small value makes it difficult to miniaturize certain types of wearable and stretchable antennas such as the microstrip antenna and planar inverted-F antenna [17]. The performance of fabric materials such as cotton and polyester as the dielectric substrate in a microstrip patch antenna with conventional copper for patch and ground plane has been evaluated and the returned loss of the antenna at resonance is ~−15 to −20 dB, indicating a good radiation efficiency [18]. Replacing the dielectric substrate with a textile substrate while keeping the conventional metals for the radiation components evaluates the effect of the textile substrate on the antenna performance [15,19]. Even with the non-uniform thickness in the textile substrate, the measured resonance frequency agrees reasonably well (an error of ~6.1%) with the results obtained from the simulation that takes the assumption of a uniform thickness. 

**Figure 1:**
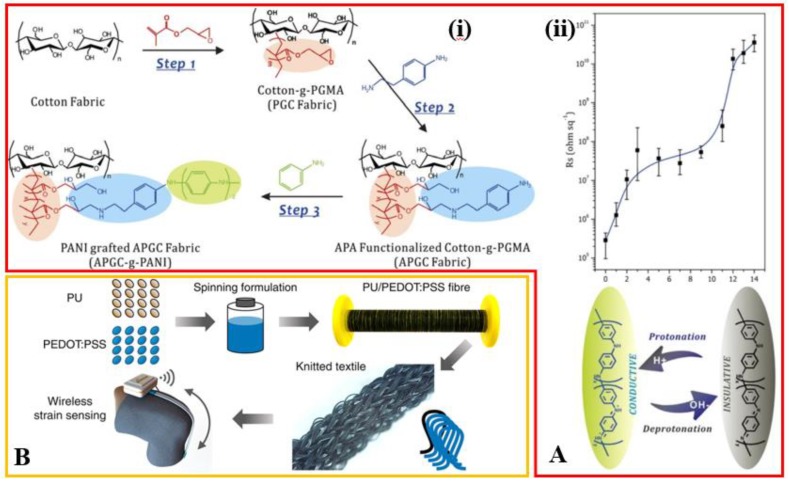

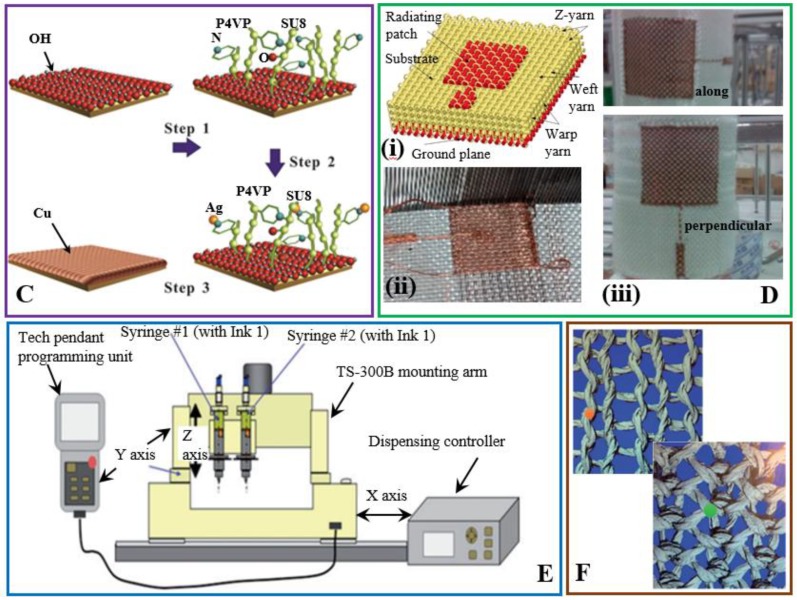
**Textile antennas.** (**Ai**) Preparation of the conductive textile by chemical grafting of polyaniline (PANI) onto cotton fabrics. PANI is linked to cotton fibers through two chemical modifications of the side chain of fibers. (**Aii**) The sheet resistance of the conductive fabric after immersing in bath solutions with different pH values. The inset schematic shows the transformation between emeraldine base (insulating form) and emeraldine salt (conductive form) by H^+^ protonation and OH^−^ deprotonation of PANI chains. Reproduced with permission from [20]; Copyright 2015, Nature Publishing Group. (**B**) The schematic illustration shows the preparation of polyurethane (PU)-based conductive fibers after mixing the solution of PU with the poly(3,4-ethylenedioxythiophene) polystyrene sulfonate (PEDOT:PSS) fillers followed by a spinning process. Reproduced with permission from [23]; Copyright 2015, American Chemical Society. (**C**) Schematic illustration of the electroless deposition of copper on cotton fabrics. Step 1: poly(4-vinyl pyridine) (P4VP) is first attached to cotton fabrics by dip coating. Step 2: silver ions are absorbed due to the strong affinity of pyridyl groups to metals. Step 3: silver ions are reduced to silver particles, which will act as the catalyst for the copper deposition. Reproduced with permission from [36]; Copyright 2016, Royal Society of Chemistry. (**Di**) Schematic and (**Dii**) weaving process of the 3D fabric antenna. (**Diii**) Optical images show the bending of 3D fabric patch antennas with the curvature along with or perpendicular to the feeding direction. Reproduced with permission from [39]; Copyright 2016, SAGE Publications. (**E**) Patterning of conductive traces on textile substrates with an automatic dispensing system by sequent dispensing of copper sulfate (CuSO_4_) and sodium borohydride (NaBH_4_). A conductive copper film forms after the redox reaction. Reproduced with permission from [40]; Copyright 2014, SAGE Publications. (**F**) Micro-computed cosmography images of conductive fabrics with different knitted structures. The contact resistance of the top (plain knit, the interlock of the knit stitches) is contributed by the overlap of two conductive yarns, whereas that of the bottom (single pique with 50% tuck stitch, the interlock of the knit stitch and the tuck stitch) is from three conductive yarns. The latter one with more conductive pathways has a smaller contact resistance. Reproduced with permission from [41]; Copyright 2018, SAGE Publications.

In order to provide an all-textile antenna, the radiation components need to be replaced by conductive fabrics as well. Various methods have been explored to obtain conductive fabrics, including chemical modification and physical mixing the fabric with conductive components. In one attempt to chemically modify the fabric surfaces, polyaniline (PANI) is covalently grafted onto a fabric substrate to yield a conductive fabric (Figure 1(Ai)) [20]. Post-treatment in bath solutions of different pH values can further tune the conductivity of the fabric, which provides a switch between conductive (sheet resistance of ~2.5 × 10^5^ Ω/□ at pH of 0) and insulating (sheet resistance of ~4 × 10^10^ Ω/□ at pH of 14) behaviors (Figure 1(Aii)). The decrease in the pH of the reactant solutions results in an increase in the degree of protonation (H^+^ doping), leading to a decrease in the sheet resistance of the fabric. In contrast, OH^−^ deprotonation of PANI chains in the reactant solution with high pH levels leads to an increased sheet resistance. The electrically conductive property of the resulting fabric is also shown to be highly stable. As demonstrated in the simulated dry-wash test, the conductivity of the conductive fabrics with different degrees of grafting (DG) of PANI shows negligible changes after 40 times of dry-wash cycles. Even though the conducting fabric has the merit of softness and washability, the relatively high sheet resistance would lead to a poor antenna performance such as a radiation efficiency of less than 10% [21,22].

The physical mixing involves the use of several conductive materials. As a widely used organic conductive material, poly(3,4-ethylenedioxythiophene) polystyrene sulfonate (PEDOT:PSS) can be mixed with the solution of polyurethane (PU) to modify its conductivity in the spinning process (Figure 1B) [23]. For instance, mixing the PU solution with PEDOT:PSS 13.0% (weight ratio) yields a conductive composite fiber with an electrical conductivity of 940 S/m and a stretchability of 345%. Mixing carbon nanotubes (CNTs) with the polymer has attracted great attention because of the reinforced mechanical and electrical properties [24,25,26,27,28,29,30]. In order to explore CNTs in the conductive fabric, coating textile with a mixed solution of aqueous CNT dispersion and a water-based polyacrylate dispersion binder can lead CNT particles to form a honeycomb structured conductive network in the coating [24], which results in a sheet resistance of ~60 Ω/□. These conductive fabric materials with improved conductivity have great potentials in the application of wearable antenna [31,32].

In order to improve the electrical conductivity, metal plating and especially the electroless plating on fabric has been explored [33,34,35]. Among a variety of metals (e.g., nickel, silver, and copper) that have been explored, the silver-plated fabric is found to have the best electrical conductivity due to the ultrahigh conductivity of silver [33]. With a comparable conductivity but at a much lower cost, the copper plating is widely used. In a typical process of the copper-plated fabric, cotton fibers are firstly modified with poly(4-vinyl pyridine) (P4VP) to serve as an adhesive layer for silver ions (Figure 1C) [36]. Next, Ag obtained after reduction of silver ions acts as a catalyst for the subsequent Cu deposition in the electroless coating bath. The conductivity of the resulting textile initially increases with the increase of the P4VP concentration and then saturates because of a poor fluidity and non-uniform coating, yielding a maximized sheet resistance of ~0.04 Ω/□. 

It is of significant interest to precisely control the size of radiations parts in the textile antenna. Among the different approaches, laser cutting, programmable knitting or embroidery, and printing are widely adopted. In the programmable embroidery, an embroidery design program first generates digitalized patterns for the sewing machine. Precisely embroidering flexible silver-coated fibers in a double-layer manner onto a regular fabric, assembled onto the polymer substrate, yields basic RF prototypes such as transmission lines, patch antennas, and antenna arrays with comparable performance with their copper counterparts [37]. The precision of embroidery can reach ~0.1 mm, promising great potential in the industrial application [38]. In another study, weaving copper yarns (conductivity of 10^7^ S/m) as a radiating patch and the ground layer with E-glass fibers of five layers as the substrate in the 3D fabric antenna is achieved by a 3D orthogonal weaving machine (Figure 1D) [39]. When the antenna is bent with the curvature along with the feeding direction, the effective resonant length decreases, leading to an increase in resonance frequency. In comparison, the antenna with a bending curvature perpendicular to the feeding direction shows a relatively stable resonance frequency. Printing technique represents an alternative to precisely control the size of the pattern. As shown in Figure 1E, copper sulfate (CuSO_4_) and sodium borohydride (NaBH_4_) solutions can be sequentially dispensed through two syringes [40]. After oxidation and reduction, a uniform conductive copper layer forms on the textile surface. However, the wettability of textiles could pose a potential issue for a uniform coating of copper.

The sheet resistance of the textile also depends on the design and process of knitting or weaving. As shown in Figure 1F, the conductive fabric in the single pique structure (orange dot in the bottom one) with more conductive pathways has a smaller contact resistance than the plain knit structure (red dot in the top one) [41]. As the pliability of the conductive fabric is of critical importance to the textile antenna in the practical applications, the mechanical properties of the base fabric need to be closely examined as well. Due to the knitting structure in the fabric, its mechanical properties are intrinsically anisotropic. The Young’s modulus of fabrics ranges from tens of kPa to several MPa and the Poisson ratio is in the range of [0.1, 0.4] [42]. The specific techniques to assemble the antenna with a fabric substrate and conductive fabric radiation part also need to be carefully chosen, as the electrical short or additional loss may occur [17]. The widely used methods include a connection with a seam, a thermal adhesive layer, and silicone encapsulation. 

Due to the soft mechanical properties of the fabric, textile antennas can easily be bent without the loss of function, as demonstrated in the operation of microstrip [43] and dipole [44] antennas. Likely due to the negligible change in the length of radiation parts, the resonance frequency of the textile antennas remains almost unchanged during the bending. Though flexible, the textile is not intrinsically stretchable. Thus, a structural design has been explored to enable its stretchable properties. In the cases of monopole and dipole antennas that are most widely used because of their simple structure and ease of fabrication, the arm can be designed into serpentine structures embedded in an elastomer matrix [45,46]. With a negligible elongation in the length of the antenna arm upon a tensile strain, a relatively stable resonance frequency can be expected. When embedded into a tire, a stretchable dipole antenna with silver-coated fabrics as the two serpentine arms and PDMS for the encapsulation layer has a longer operation range (2.8 m) than a commercial antenna (1.2 m). Combining such an antenna with the sensor, RF chip, and microcontroller would present the promising potential for precise and stable tire monitoring (e.g., tire revolutions and pressure) [45].

## 3. Composite Elastomer with an Interpenetrating Network of Liquid Metal

It represents an interesting direction to fill the microfluidic channel created from elastomeric polymers with room-temperature liquid alloys. The ability to flow liquid metal in the microfluidic channel ensures electrical continuity even under various loading conditions. The fairly simple and scalable process does not involve etching or plating that produces hazardous waste. It can also be easily integrated with other 2D/3D devices and especially fluidic components for sensing and actuation [47,48]. Compared with mercury, gallium alloy is more widely used due to its nontoxicity and good conductivity (3.46 × 10^6^ S/m) [49]. In addition to being used for stretchable interconnection [50,51,52], the design has attracted increasing attention in stretchable antennas [53] and metamaterials [54]. The demonstrated examples include a stretchable unbalanced loop antenna [55,56], a half-wave dipole antenna [57,58], a patch antenna [59,60], and a planar inverted cone antenna [61]. 

**Figure 2:**
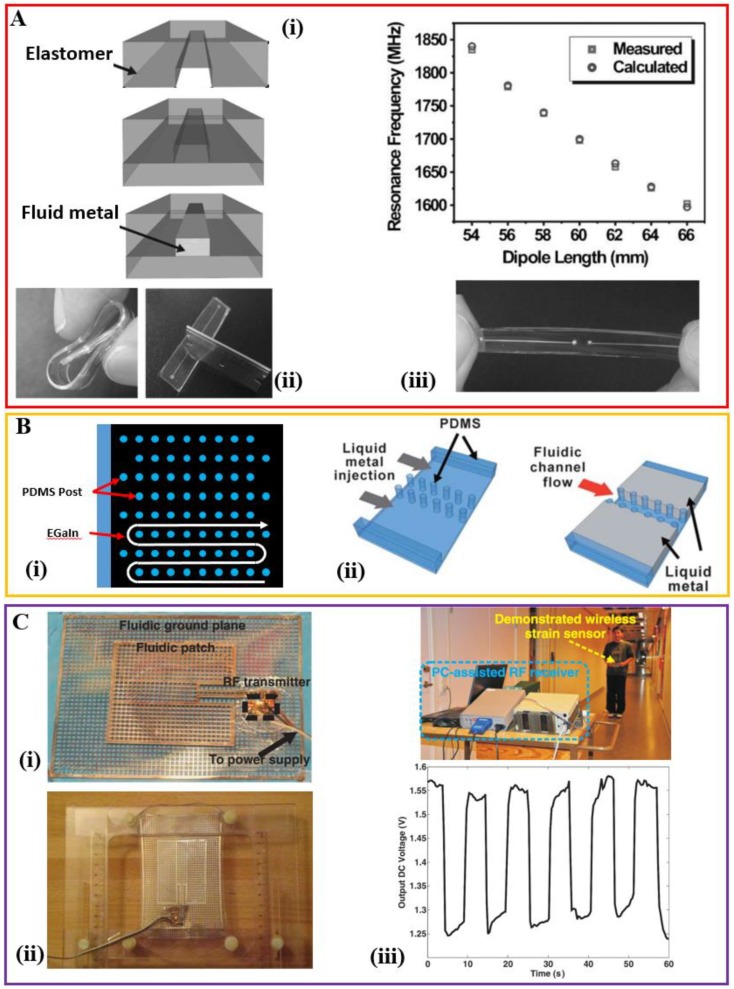
Stretchable microfluidic antennas filled with the liquid metal. (**Ai**) Schematic illustration of the molding and injection process to fabricate the liquid metal-based stretchable dipole antennas. (**Aii**) Demonstration of the deformability and self-healing capability. (**Aiii**) The measured resonance frequency of the dipole antenna with stretching. The experimental measurements show an approximately linear relationship. Reproduced with permission from [58]; Copyright 2009, John Wiley and Sons. (**Bi**) Schematic illustration of the patch composed of serpentine microfluidic channels defined by an array of posts. (**Bii**) Separated by two parallel rows of PDMS posts, liquid metal microelectrodes are created to be in direct contact with the central fluidic channel for applications such as electrohydrodynamic mixing and dielectrophoresis. Reproduced with permission from [62]; Copyright 2011, Royal Society of Chemistry. (**C**) The stretchable patch antenna with the meshed ground plane and patch of the liquid metal (**Ci**) before and (**Cii**) after stretching. (**Ciii**) shows a wireless strain detector based on the stretchable patch antenna. The output voltage of a receiving horn antenna changes with the stretching state of the transmitting antenna because of the resonance frequency shift. Periodic stretching and releasing lead to a periodic change of the output voltage in the receiving antenna. Reproduced with permission from [59]; Copyright 2011, John Wiley and Sons.

The unbalanced loop antenna with a resonance frequency of 2.4 GHz exhibits a stretchability up to 40% along two orthogonal orientations, with a radiation efficiency of over 80% [56]. The resonance frequency of the half-wave dipole antenna can be tuned in a linear way by mechanically stretching the antenna without hysteresis upon relaxation, useful for the wireless strain sensing (Figure 2A) [58]. The antenna also self-heals in response to sharp cuts such as those induced by a razor blade and returns to its original conducting state, which is possibly due to the elasticity of the PDMS. In the case of a microstrip patch antenna, a complete, evenly filled liquid metal is required in the co-planar sheet-like geometries (in both patch and ground plane). In order to shape the liquid metal in such requisite geometry, a serpentine pathway of posts with constant height has been designed (Figure 2(Bi)) [60]. Following the Young-Laplace equation that governs the minimal pressure to induce the flow, a careful design in the spacing between adjacent posts in the array can guide the liquid metal to flow in the desired serpentine pathway. An appropriate height of the posts can also prevent the channel collapse. Taken together with the fact that a thin oxide skin spontaneously and rapidly forms on the surface of the liquid metal, the idea of periodic posts also allows the creation of microelectrodes of liquid metals to have direct contact with the fluid in the adjacent microfluidic channels for many microfluidic applications such as electrophoresis (Figure 2(Bii)) [62]. 

An alternative to the use of periodic posts, a meshed structure is also explored to shape the plane geometry [59,63]. The resonance frequency of the resulting stretchable antenna decreases with stretching up to 15% before mechanical failure due to the mechanical mismatch. Due to the shift of resonance frequency of the stretchable antenna under stretching, it presents a mismatch in the resonance frequency to the receiving horn antenna, consequently leading to the decrease of the received power (or output voltage) by the horn antenna (Figure 2C). This stretchable antenna based on a liquid metal mesh network can work as strain detectors in a wireless mode with a very low power consumption. A mechanically flexible planar inverted cone antenna (PICA) has also been demonstrated for ultra-wideband (UWB) application [61]. The presented antenna has a radiation efficiency of over 70% in the range of 3–10 GHz (a return loss better than 10 dB within 3–11 GHz) and it also allows a tensile strain up to 40% along either x- or y-direction. 

**Figure 3:**
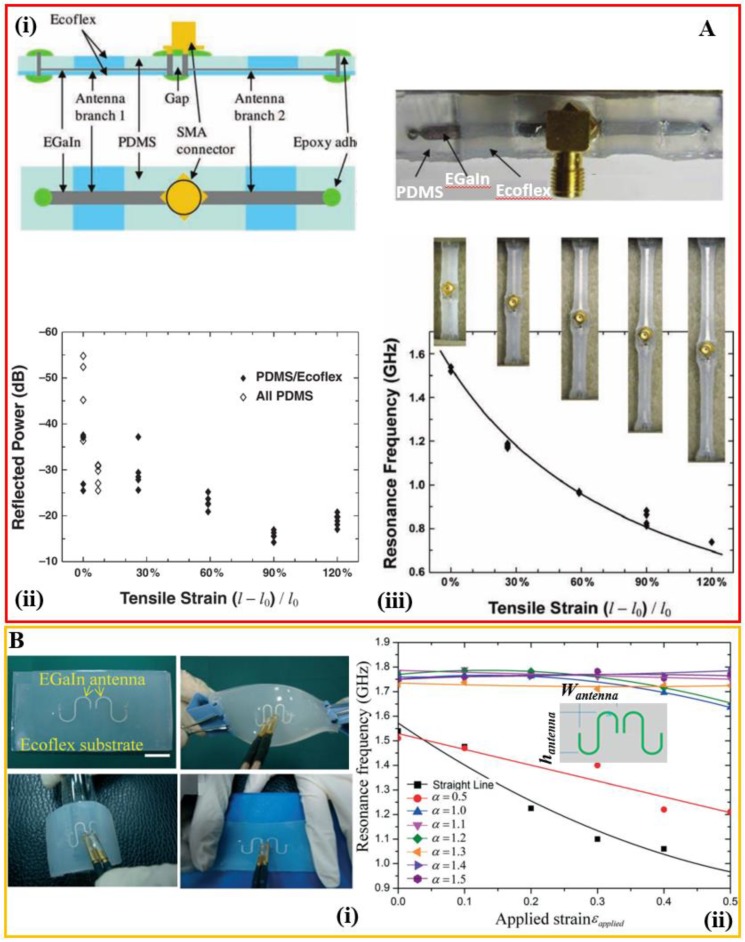
**Structural design of the microfluidic antenna filled with the liquid metal** (**Ai**) Schematic and optical image of the structural design used to improve the stretchability of the dipole antenna that is based on the liquid metal. (**Aii**) Resonance change as a function of a tensile strain. (**Aiii**) Comparison of the radiation efficiency of the antenna with (i.e., PDMS/Ecoflex) or without (i.e., all PDMS) structural engineering under various levels of tensile strain. Notably, failure of the “all-PDMS” structure occurs for a tensile strain of 20%. Reproduced with permission from [57]; Copyright 2010, John Wiley and Sons. (**Bi**) Optical images of the microfluidic dipole antenna filled with the liquid metal with two serpentine arms in an undeformed state, and being twisted, bent, and stretched. (**Bii**) The resonance frequency change as a function of the tensile strain. Depending on the specific aspect ratio (i.e., the ratio of nominal height *h_antenna_* to width *W_antenna_*), the microfluidic antenna can be designed to be strain-dependent or independent. Reproduced with permission from [64]; Copyright 2014, Royal Society of Chemistry.

The stretchability of these demonstrated systems is significantly less than the maximum strain that the elastomer can sustain. The reason is possibly arising from the fact that the uniform stretching in a single type of elastomer would lead to breaking at weak points: (1) inlets and outlets of the microfluidic channels, and (2) interfaces between the elastic and rigid parts such as the external electrical connectors of the devices. To address this challenge, a hybrid design integrates silicone polymers with different stiffness to build the microfluidic channel, where a stiff elastomer such as PDMS is used in the regions with weak points and a softer elastomer such as Ecoflex is used to enhance the stretchability in the other regions (Figure 3A) [57]. The demonstrated half-wave dipole antenna is highly stretchable (i.e., functional over a tensile strain of 120%) with a wide tuning range that follows an inversely proportional relationship between the resonance frequency (*f* in MHz) and the length of the antenna (*l* in m): $f=143/\left(l\sqrt{\varepsilon_{eff}}\right)$ where $\varepsilon_{eff}$ is the effective dielectric constant of the medium. The radiation efficiency of the antenna is over 95% even at a tensile strain of 120% and a reliability test also demonstrates negligible changes (i.e., within 1%) in the resonance frequency and radiation efficiency with the antenna being stretched 100 times for a tensile strain of 50%. Another technique to address the issue of a mechanical mismatch for improving the stretchability is to use the slot-aperture-coupled feeding technique, where the rigid feed line is separate from the stretchable patch [63].

While it is useful to have a wide tuning of the resonance frequency in the designed stretchable antenna, it is also highly desirable for the stretchable antennas to have unaltered resonance frequency upon stretching, which may open up opportunities for easy transmission of data or energy in bio-integrated electronics. Because the conductive element of the antenna is the fluidic liquid metal, the shape and mechanical properties of the antenna are defined by the elastomeric channels. As the resonance frequency of a half-wave dipole antenna upon stretching primarily depends on the deformed length of the antenna, rational design of the antenna would give the same deformed length as the initial length. 

One design shapes the dipole antenna in a serpentine geometry with a specific aspect ratio (i.e., the ratio of nominal height *h_antenna_* to width *W_antenna_*) to provide an almost unaltered resonance frequency for a tensile strain up to 50% (Figure 3B) [64]. Depending on the aspect ratio, the resonance frequency can also increase or decrease in a tunable manner, but the design with an increasing resonance frequency requires a large aspect ratio in the antenna that is associated with a weak electrical signal for the measurement. The almost unaltered resonance frequency with the tensile strain is currently limited to the stretching in one direction; thus, it is desirable to have a design for omnidirectional unaltered resonance frequency.

## 4. Composite Elastomer Embedding Conductive Fillers

Although the relationship between the antenna performance and the conductivity especially during stretching for a stretchable antenna is not explicitly studied, it is believed that a high conductivity during stretching is of critical importance. A significant reduction in conductivity is commonly observed in polymer composites loaded with conductive fillers under tensile strain [65]. The phenomenon (i.e., piezoresistive effect) is a result of filler displacement or rotation within the matrix. The resistive response to the strain of composites determines their practical applications. Composites with a large piezoresistance have been widely studied in the strain sensing [66,67,68,69]. Analytical models of the piezoresistance of polymer composites with conductive fillers such as carbon black [70,71], CNTs [30,72], and metal spheres [73] indicate that the conductivity $\sigma_{stretched}$ upon stretching is related to its initial conductivity $\sigma_{initial}$ as:

(1) $$\sigma_{stetched}\approx \sigma_{initial}\exp\left[\frac{4\pi \sqrt{2m\phi }}{h}\left(s_{initial}-s_{stetched}\right)\right],$$

where *m* is the mass of an electron, ϕ is the height of the tunneling potential barrier, *h* is Plank’s constant, and $s_{initial}$ (or $s_{stretched}$) are the average interparticle distance between fillers before (or after) stretching. In order to maintain a high conductivity during stretching, it is highly desirable to reduce the tunneling potential barrier ϕ or to minimize the interparticle distance $s_{stretched}$ during stretching. The strategy toward the former could include removal of the insulating lubricant on the surface of the fillers. The desired properties toward the latter could be enabled by the careful design of the shape and morphology of the fillers such as a high aspect ratio. Mixing silver nanoparticles with PDMS shows a relatively high conductivity of 1000 S/cm and the value is observed to increase slightly upon a tensile strain of 30% [74]. Though a high silver volume fraction in the composite yields a high conductivity and a stable conductivity change over a tensile strain, its high viscosity also makes it difficult to print. The dipole antenna prepared by a standard stencil printing of the Ag-PDMS composite is demonstrated to have a low loss and long distance communication capability in the on-body scenario. When compared with nanoparticles, the nanowires with a high aspect ratio show a more stable performance in the conductivity over a tensile strain. For the highly aligned fillers, the high conductivity is only maintained for a stretching along the aligned direction [75,76]. Although randomly dispersed 1D fillers can provide better omnidirectionally high conductivity under a small tensile strain, they begin to align along the stretching direction and lose the conductive path in the other two directions, resulting in a decrease in the overall conductivity [77]. 

**Figure 4:**
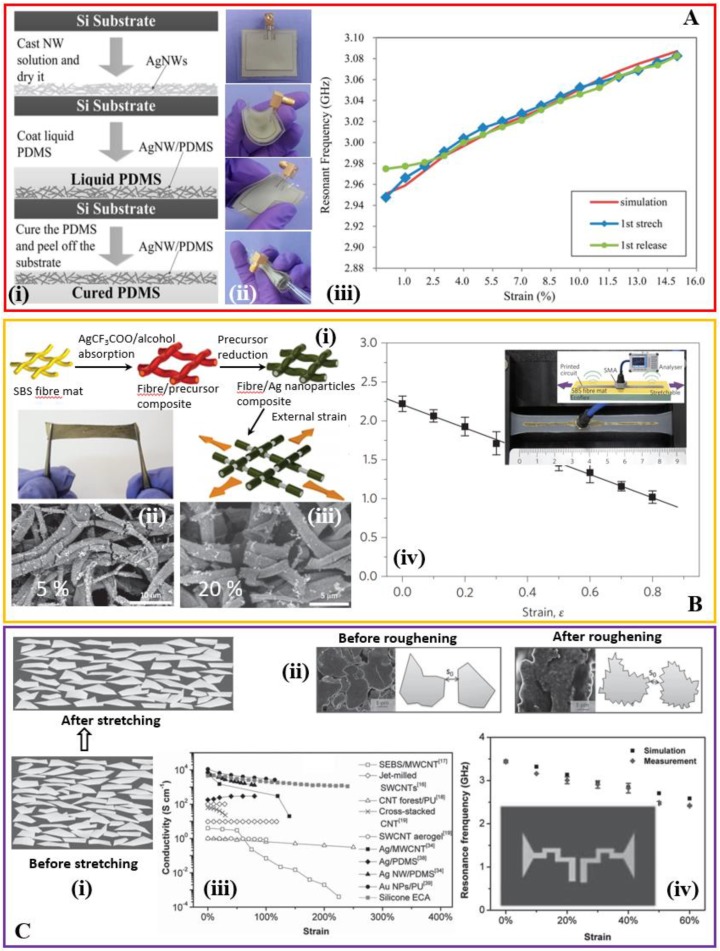
**Stretchable antenna based on composite elastomers embedding conductive fillers.** (**Ai**) The fabrication process of the stretchable patch antenna with the AgNW-PDMS composite. AgNW: silver nanowire. (**Aii**) The stretchable patch antenna in the undeformed and various deformed states such as bending and twisting. (**Aiii**) The measured and simulated resonance frequency of the antenna as a function of the tensile strain. Upon stretching and releasing, no obvious hysteresis is observed. Reproduced with permission from [79]; Copyright 2014, American Chemical Society. (**Bi**) Schematic illustration of electrospun poly (styrene-block-butadiene-block-styrene) (SBS) rubber fibers with interconnected silver particles inside the fibers and on the surface to act as conductive pathways. The composite of the SBS and silver particle is highly conductive even at a tensile strain of 100%. SEM images of the composite at a tensile strain of (**Bii**) 5% and (**Biii**) 20%. Even with silver debris at a tensile strain of 20%, only a slight decrease in the conductivity is observed. (**Biv**) Change in the resonance frequency of a dipole antenna that uses the conductive composite as the two radiation arms (inset). Reproduced with permission from [80]; Copyright 2012, Nature Publishing Group. (**Ci**) Schematic illustration of a conductive composite consisting of silicone polymer and silver flakes. (**Cii**) SEM images and schematic illustrations that show the roughening process of the silver flakes by using long-chain hydride-terminated polydimethylsiloxane (H-PDMS), leading to a decrease in the interparticle distance between neighboring flakes. (**Ciii**) When compared with various previous studies, the silicone-based electrically conductive adhesive (silo-ECA) only shows a slight decrease in the conductivity for a tensile strain of 100%. (**Civ**) Change in the resonance frequency of a bow-tie antenna that uses the conductive composite as radiation components as a function of tensile strain. Reproduced with permission from [81]; Copyright 2015, John Wiley and Sons.

Silver nanowires (AgNWs) are a class of representative 1D fillers used in many stretchable electronic devices. AgNWs synthesized via a copper (II) chloride (CuCl_2_)-mediated polyol process is first deposited and etched into a patterned geometry. Casting and curing of a PDMS layer result in a composite layer with AgNW in the top surface of PDMS. The patch and the ground plane prepared with the same process is then bonded to form a patch antenna [78]. When subject to a pressure applied on the top surface of the patch antenna, its resonant frequency decreases from 2.37 GHz to 2.27 GHz as the force (or pressure) increases from 0 to 10 N. A transmission line fabricated with the same process also only shows a slight change in the S-parameters upon bending over a bending radius of 10 mm. Although a direct tensile test is not conducted in this study, the resulting antenna is expected to deform for an applied tensile strain. In a similar study, casting and curing a liquid PDMS on top of the screen printed AgNWs conductive film result in the conductive AgNW film embedded as a surface layer in the PDMS substrate (Figure 4A). Bonding the patch layer with a ground plane layer yields a 3-GHz microstrip patch antenna and a 6-GHz 2-element patch array that are mechanically tunable and reversibly deformable. The resonance frequency approximately shows a linear change with the applied tensile strain, so it could be envisioned for applications such as wireless strain sensing [79]. 

Beside nanowires, a conductive composite mat of silver nanoparticles and rubber fibers has been exploited to achieve a high conductivity during stretching because of the formation of the conductive silver network in the rubber fiber (Figure 4B) [80]. Reducing the silver nanoparticle precursor absorbed in the electrospun poly (styrene-block-butadiene-block-styrene) (SBS) rubber fibers yields a mat with percolated silver nanoparticles inside the fiber. The fiber mat shows a high bulk conductivity even at large deformations and the conductivity decreases from ~5000 S/cm from the initial state to ~2200 S/cm at a tensile strain of 100%. Although the upper surface of the fiber mat from electrospinning on a silicon wafer is porous with pores of a few micrometers, its surface roughness can be greatly reduced by sandwiching the fiber mat between two silicon wafers followed by a thermal annealing at 90 °C for a short time of 10 min. With a slight decrease from 150 μm to 120 μm in the thickness, the average surface roughness of 134 nm is obtained, allowing direct printing of the precursor solution through conventional techniques such as nozzle printing or inkjet printing. Direct chemical reduction of the precursor solution results in electrical conduction with applications in the stretchable radiofrequency antenna, strain sensors, and other circuit components. The stretchability of the prepared half-wave dipole antenna allows tunability over a wide range of frequencies with a similar level of high-quality radiation efficiency.

In another effort to achieve high conductivity in the highly deformed state, the iodine treated silver flake is mixed with the silicone mixture matrix in a weight ratio of 80:20 to result in a silicone-based electrically conductive adhesive (silo-ECA) (Figure 4C) [81]. Two surface modification methods have been used to form a strong conductive network: (1) reduction of the coordinated silver salt in the commercial silver flakes by using long-chain hydride-terminated polydimethylsiloxane (H-PDMS) and (2) exposure of fresh silver at the flake surface by iodination treatment. After the modification, the electrically insulating lubricant layer on silver flakes is removed and the silver flake develops into a spike-like shape due to the chemical reduction. The combined surface modification method helps reduce the tunneling potential barrier ϕ between the silver flakes and the inter-tunneling distance to yield a high conductivity before and after stretching (e.g., the initial conductivity of 1.51 × 10^4^ S/cm and a conductivity above 1.11 × 10^3^ S/cm upon a tensile strain of 240%). In addition, the shear force during the printing process allows the silver flakes to stack parallel to one another, which ensures that the distance between flakes is almost unaltered during stretching in a certain range. By using the stretchable silo-ECA as the conductive pattern and the pure silicone polymer as the substrate, stretchable circuits can be fabricated through the soft-lithography process or stencil printing. The resulting quarter-wavelength bowtie antenna shows a tunable resonant frequency with high-quality radiation efficiency and almost a linear dependence on the tensile strain. It should be noted that the antenna could be stretched over the demonstrated 60%, but the weak connection with the SMA connector likely causes the mismatch for damage. It should also be noted that besides the examples discussed above, the recent studies on the highly stretchable, conductive composite elastomers also show great potential in stretchable antennas. The representative examples include an all-polymer stretchable conductor by doping ionic additive–assisted enhancers in PEDOT:PSS [82], printable elastic conductors formed in situ by mixing Ag nanoparticles with micrometer-sized Ag flakes [83], the biocompatible Ag-Au core-sheath nanowire [84], among others.

## 5. Stretchable Structures from Conventional Metals

By avoiding localized plastic deformation such as local thinning or forming shear bands [85], thin films of gold evaporated onto elastomeric membranes of polydimethylsiloxane (PDMS) allow the system to be stretched over tens of percent that far beyond the fracture strain of a freestanding gold film [86,87,88,89]. Applying the concept of stretchable metals as conductive elements in the design of antennas yields stretchable antennas with conventional metals. The stretchable planar inverted-F antenna (PIFA) can operate under a tensile strain up to 10% with a reversible performance when relaxed to 0%, but it shows a relatively poor efficiency with an extra 10 dB loss compared with a standard one, likely due to the thin geometry and low conductivity in the evaporated Au film [90]. Therefore, ink-jet printing has been used to produce thick (200–400 nm) silver films on a patterned PDMS substrate for a dipole antenna. Comparing with a standard dipole antenna, the stretchable dipole antenna shows an extra loss of 1.7 dB and its performance is not reversible. 

**Figure 5:**
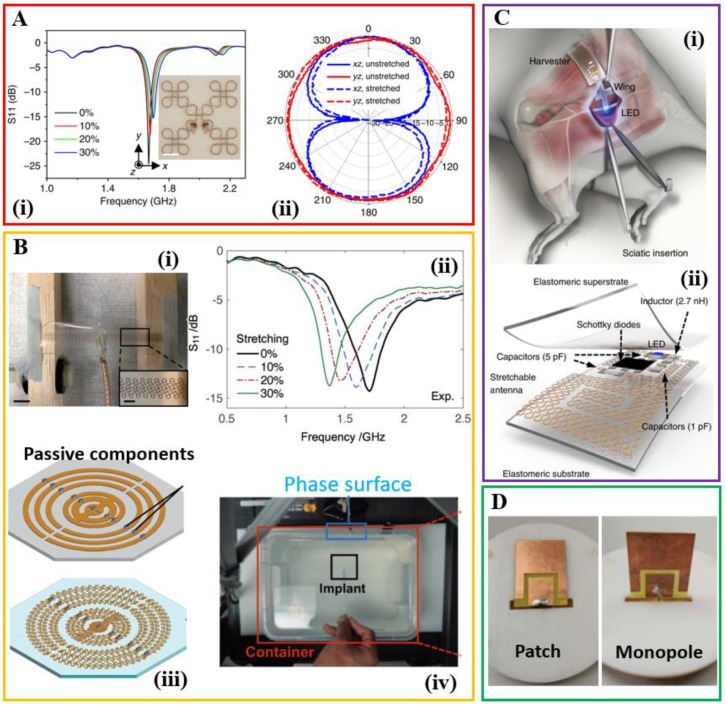

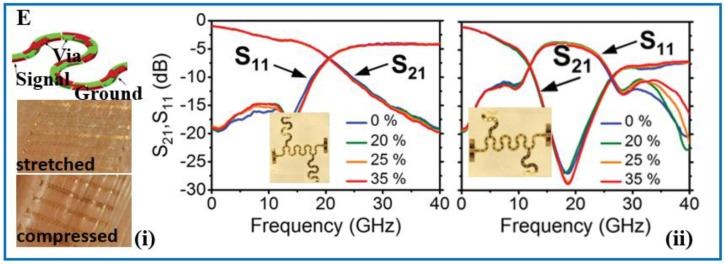
Stretchable antennas that exploit stretchable structures of conventional metals. (**Ai**) Return loss parameters of a fractal Vicsek curve loop antenna under different levels of tensile strain. The inset shows an optical image of an unstrained antenna fully bonded onto an elastomeric substrate. Scale bar, 4 mm. (**Aii**) Far-field profiles of the loop antenna only show a slight change in the radiation patterns at 30% tensile strain. Reproduced with permission from [92]; Copyright 2014, Nature Publishing Group. (**Bi**) Stretchable dipole antenna by converting solid radiation parts to serpentine mesh layouts. (**Bii**) The corresponding reflection curve with an increasing tensile strain. (**Biii**) Transformation of a midfield phased surface from a solid structure to a meshed stretchable layout. (**Biv**) demonstration of powering an LED with the stretchable midfield phased surface at different levels of tensile strain. Reproduced with permission from [14]; Copyright 2017, John Wiley and Sons. (**Ci**) Anatomy and location of the peripheral and epidural devices relative to the sciatic nerve. (**Cii**) Exploded view schematic illustrating the energy harvester component that includes the stretchable antenna, AC/DC converter, and an integrated LED toward wireless optogenetics. Reproduced with permission from [95]; Copyright 2015, Nature Publishing Group. (**D**) Photograph of fabricated antenna that can be mechanically reconfigured from a patch state to a monopole state. Reproduced from [101]; Copyright 2017, John Wiley and Sons. (**Ei**) Stretchable twisted-pair transmission line inspired by twisted-pair cables. (**Eii**) Scattering (S-) parameter of a stretchable low-pass filter (**left**) and band-stop filter (**right**) at a tensile strain of 0%, 20%, 25%, and 35%. The inset shows the corresponding optical image of the two filters. Reproduced with permission from [102]; Copyright 2016, John Wiley and Sons.

As stretchable structures have been widely exploited to enable stretchable properties to conventional brittle materials toward stretchable electronics, it occurs naturally to apply such concept in the design of the stretchable antenna, where conventional metals can be used as conductive elements. As briefly discussed in the stretchable textile antenna, antennas with simple geometries (e.g., monopole and dipole antennas) can be easily designed into a stretchable form by replacing the straight lines with serpentine lines. As an alternative to the stretchable material, meandered structures provide stretchable properties to enable a stretchable antenna. A meandered dipole with a parasitic arm made of copper on the silicone and polyurethane (TPU) substrate has demonstrated a wideband match in stretched conditions, with a reflection coefficient lower than −10 dB up to a stretching of 20% at the designed 2.4 GHz working range [91]. On-body measurement with physical phantoms also shows the practical values of the reflection coefficient and radiation efficiency when there is a separation of a few millimeters between the antenna and the human body. Photo-lithographic processes also allow the creation of patterned copper foils for fractal antennas and a demonstrated example shows a Vicsek curve loop antenna with a fundamental mode near 1.7 GHz and an impedance of 42 ohms (Figure 5A) [92]. When mounted on stretchable substrates, the fractal antenna offers not only a compact nature (total length of λ_0_/6 at resonance with λ_0_ of the free space length), but also stretchable characteristics (almost unchanged resonance frequency and radiation patterns upon a tensile strain of 30%). 

When the geometry becomes complicated, strategies have been explored to convert a rigid metal section with a curvilinear layout to a mesh with rectangular and trapezoidal unit cells, and then to a serpentine mesh layout [14]. Implementing the strategy to a microstrip transmission line shows the change of effective electrical lengths in the design with different arc angles in the serpentine mesh. The attenuation constant and phase constant are also shown to depend on the arc angle. Based on serpentine mesh layouts, a natural tradeoff between the stretching mechanics and microwave performance of the system is observed. Changes of effective wavelength and power attenuation in microstrip transmission lines with a serpentine layout are also observed in other metallic structures. In the case of a far-field dipole antenna, these two changes correspond to the changes in resonance frequency and antenna gain (or radiation efficiency), respectively (Figure 5B) [14]. A complicated geometry such as a midfield phased surface with concentric metal rings has also been demonstrated to focus microwave energy for powering implanted biomedical devices (e.g., light-emitting microdevice) inside the human body (through water that mimics biological tissue). Because a thicker trace is used in the midfield phased surface, the stretchability is limited to 10% and the efficiency only decreases by 2.2 dB under the maximal stretching. A similar serpentine geometry but with a freestanding design has also been applied to a copper/polymer thin film bilayer for a monopole antenna [93]. Even after a tensile strain of up to 30% and for 2000 cycles, the antenna still retains the essential properties, including resonance frequency and bandwidth, gain, radiation pattern, and directionality. In addition, the transmitter board integrated with the stretchable antenna has shown the capability for the far-field communication. With the stretchable antenna mounted on a stretchable fabric and worn by a human subject, the RF power of 1 dBm (1.25 mW) from the transmitter reduces to −100 dBm over a distance of 80 m in an open area on the university campus [93]. When combining the optogenetics with the stretchable antenna that harvests near or far field RF power between adjacent serpentine traces [94,95,96,97], the integrated system achieves optogenetic modulation of the spinal cord and peripheral nervous system (Figure 5C) [95]. This device would facilitate the long-period experimental study on neuronal circuitry. 

The concept of deformable structure can also be used for the design of reconfigurable and deployable antennas [98,99,100]. For instance, the resonant frequency of the antenna can be tuned over a wide range of distinct and discrete frequencies with robotic growth (i.e., tip-extending) that is controlled by a closed-loop system [98]. The demonstrated examples include monopole, Yagi-Uda, and helical antennas. In a similar idea, rotating the top substrate mechanically reconfigures a dual-band antenna from a patch state (with an omnidirectional radiation pattern) to a monopole state (with a directional pattern) (Figure 5D) [101]. Combining such concept with smart materials such as those responding to external triggers could further enhance the performance of the system. The idea of stretchable structures can also be applied from antennas to transmission lines such as those that are capable of delivering microwave signals (beyond the previously reported direct current or low-frequency signals). A twisted-pair transmission line integrated into thin-film serpentine microstructure minimizes electromagnetic interference to allow its use in bioelectronics (Figure 5E) [102]. The twisted-pair structures also demonstrate their use as passive components such as stretchable microwave low-pass filter and band-stop filter. From the measured S_11_ curves, it can be concluded that a stretching up to 35% does not lead to their performance degradation. With the potential use for high-speed digital circuits, this type of high-performance transmission lines could be integrated with active devices to provide high-speed wireless communication systems for remote monitoring of patients in the clinical applications.

## 6. Conclusions and Future Perspectives

### 6.1. Conclusions

In this mini-review, we have discussed several representative strategies to enable the flexible and stretchable antennas for the bio-integrated electronics. These strategies include the use of textile, liquid metal, composite elastomer with conductive fillers, and structural design of conventional materials. Despite the significant advancement in each strategy, amble opportunities still exist for future development. As tradeoff typically exists between the stretchable mechanical property and the antenna performance, new materials, novel designs, innovative approaches in the fabrication, or a synergistic combination of them to strike a balance or even go beyond the existing limit would remain an active topic in the future studies. 

### 6.2. Future Perspectives

**Figure 6:**
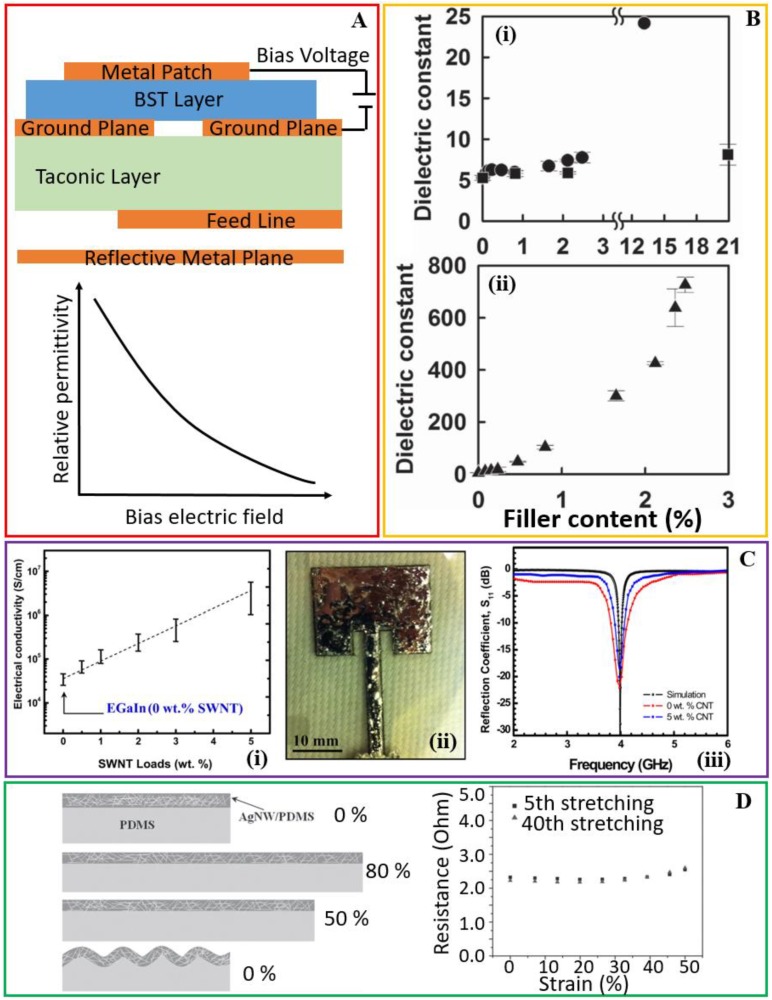
**Future directions for the stretchable antenna.** (**A**) (**i**) Applying a bias voltage between the patch and ground plane changes the effective dielectric constant of the (Ba, Sr)TiO_3_ (BST) substrate, yielding a frequency-configurable patch antenna. (**B**) The dielectric constant of the nanopaper composite at 1.1 GHz as a function of different filler content (volume ratio) of (**Bi**) BaTiO_3_ (square), Ag particles (circles), and (**Bii**) Ag nanowires. Reproduced with permission from [11]; Copyright 2015, John Wiley and Sons. (**Ci**) The conductivity of the EGaIn/SWNTs composite as a function of the nanotube loads (weight ratio from 0% to 5%). SWNTs: single-wall carbon nanotubes. (**Cii**) Photograph of a patch antenna with the conductive composite as radiation parts on a PDMS substrate. (**Ciii**) Measured and simulated reflection coefficients for antennas with different nanotube loads. Reproduced with permission from [119]; Copyright 2013, American Institute of Physics. (**Di**) Schematic showing the deformation of the top AgNW/PDMS composite layer during the stretching and releasing process, highlighting the buckling of the AgNW/PDMS layer due to the irreversible fiber arrangement. (**Dii**) Resistances as a function of tensile strains (0–50%) for the AgNW/PDMS composite in the 5th and 40th stretching circles. Reproduced with permission from [120]; Copyright 2012, John Wiley and Sons.

Regardless of the strategy used to enable the flexible and stretchable antenna, the miniaturization of the antenna is attractive to minimize the footprint of the integrated devices. One direction is to exploit the antenna in the high-frequency regime such as V of 40–75 GHz or W of 75–110 GHz bands, resulting in the antenna with a characteristic size in the millimeter range [103]. The high-frequency operation of the antenna is also associated with additional benefits such as high-speed communication and low power consumption. As an alternative to operating at the high frequency, the other approach for downsizing the antenna is to use the substrate material with a high dielectric constant [104,105]. The typical dielectric constant of the elastomeric substrate such as silicone is between 2.5 to 3 in the GHz range [103]. Considering the high dielectric constant of CNTs [106], metal micro-/nano-particles [107,108], and certain ceramics (e.g., BaTiO_3_ [104], NdTiO_3_ [109], MgCaTiO_2_ [109], and Ba_x_Sr_1−x_TiO_3_ [105]), mixing with the elastomeric substrate with these high-dielectric powders represents a simple solution. For instance, a dielectric constant of 8 and 10.5 is achieved when the PDMS substrate is mixed with 15 vol% and 25 vol% of BaTiO_3_ powders [110]. As a ferroelectric material, Ba_x_Sr_1−x_TiO_3_ could change its dielectric constant upon polarization in the electric field. By applying a voltage between the ground plane and patch (with a DC electric field changing from 0 to 10 V/μm), the change in the dielectric constant of the substrate leads to an increase of 10% in the resonance frequency of this reconfigurable antenna (Figure 6A) [111]. 

The simple method of doping a given substrate with metallic nanomaterials can substantially increase the dielectric constant of the substrate and the content of the metallic doping has to be smaller than the conductive percolation threshold [11,112]. For instance, the dielectric constant of cellulose nanopapers increases with the increasing content of the silver nanoparticle or nanowire fillers and the increase becomes sharp near the percolation threshold (Figure 6B) [11]. The dielectric constant of the high-density cellulose nanopaper with a density of 1.3 g/cm is measured to increase from 5.3 to 24.15 when adding 13.15% (volume ratio) of silver nanoparticles as the filler, and the value further increases to 726.5 with 2.48% (volume ratio) of silver nanowires as the filler (measured at 1.1 GHz). The drastic increase in the dielectric constant with the nanowire as the filler likely attributes to the formation of microcapacitor networks with insulating cellulose nanofibers sandwiched between adjacent conductive silver nanowires [113,114,115]. Moreover, the free charges trapped at the insulating-conductive interface would also improve the dielectric constant. Though both the nanoparticles and nanowires are well dispersed, the nanowires with a much higher aspect ratio are associated with a lower percolation threshold than that of nanoparticles, thereby providing an extremely high dielectric constant [114]. Printing a V-shaped dipole antenna on the composite cellulose nanopaper with an extremely high dielectric constant downsizes the antennae by about a half but still with the same resonance frequency as the one using the original nanopaper without fillers. From the analytical equation [116], it is believed that applying the substrates with a high dielectric constant can substantially downsize the patch antenna. However, it should be noted that there still exists a tradeoff between the size and radiation efficiency of the antenna [117,118]. 

When it comes to the liquid metals, special attention should be paid to the control of its surface oxide because of its important roles in the surface energy, mechanical stability, and electrical conductivity. With a significantly lowered surface energy, an oxide layer of eutectic gallium and indium (EGaIn) from the electrochemical deposition induces the easy injection of the liquid metal in (or out) of the capillary [63]. Meanwhile, the increased mechanical stiffness in the oxide skin helps stabilize the injected liquid metal in the channels in the absence of pressure [49,60]. However, the oxide skin leads to a reduced conductivity in the liquid metal. To address the issue, the EGaIn liquid metal alloy is mixed with 5 wt% single-wall CNTs to form an EGaIn/CNTs composite, leading to an increase in the conductivity by 2 orders of magnitude (Figure 6C) [119]. As a result, the reflection coefficient of the composite patch antenna demonstrates a 10 dB improvement over the EGaIn antenna. The composite antenna is also shown to have an improved long-term performance over 12 months, possibly because the improved conductivity and stiffness allow the antenna structure to repeatedly return to its initial shape after multiple deformations.

As for the composite elastomers with embedding conductive fillers, other strategies also exist to enable the high conductivity over the large tensile strain. For instance, AgNWs embedded in the surface layer of PDMA upon stretching can undergo irreversible sliding or rotation in the PDMS matrix to result in the buckling of the surface AgNW/PDMS layer upon release. This phenomenon is similar to the pre-strain strategy used in stretchable electronics. Because of the flatting of the bucked AgNW/PDMS layer, a constant resistance is observed in the subsequent stretching (up to 50% for the case with a pre-strain of 50%) (Figure 6D) [120]. Generally speaking, the conductivity of the composite elastomers with conductive fillers is smaller than those of metal-plated conductive textiles or liquid metals. But because of their simple fabrication processes such as casting/molding [121] and the easy integration with the increasingly popular 3D printing [122,123], the conductive composite elastomers also represent a great potential in the customization for commercialization, where the new materials or processes for improving the conductivity would provide a strong boost. 

The structural design of traditional metallic materials maintains their merits such as high conductivity for a high radiation efficiency, but the current studies are limited to certain serpentine structures. Future efforts are still needed to closely examine the dependence of microwave performance such as the resonance frequency and radiation pattern on different stretchable patterns/designs of the antenna. Given the vastly large design space, theoretical guidelines are of critical importance to design the performance of the antenna for different applications.

When the wearable or on-body application is of concern, the radiation efficiency of the flexible and stretchable antenna also needs to be improved. Moreover, a comprehensive testing of the mechanical and electrical durability and stability of the antenna is also required before it can be integrated with the other components in the bio-integrated devices toward the practical applications.
